# Vitamin D Deficiency in Pediatric Oncology Patients: A Single-Center Experience in Saudi Arabia

**DOI:** 10.7759/cureus.54807

**Published:** 2024-02-24

**Authors:** Dania A Monagel, Amal O Albaity, Fatimah M Asiri, Lama N Alghamdi, Raghad A Alsufyani, Reem B Alqarni, Shaden K Bahatheq, Omaima Ahmed, Naglla Elimam, Alaa Althubaiti

**Affiliations:** 1 College of Medicine, King Saud Bin Abdulaziz University for Health Sciences, Jeddah, SAU; 2 Research and Development, King Abdullah International Medical Research Center, Jeddah, SAU; 3 Oncology, Ministry of the National Guard Health Affairs, Jeddah, SAU

**Keywords:** oncology, hematology, pediatrics, cholecalciferol, vitamin d deficiency

## Abstract

Background

There is a lack of local studies on vitamin D deficiency in children with cancer. This study aims to estimate the prevalence of vitamin D deficiency in the pediatric oncology population at King Abdul-Aziz Medical City (KAMC) in Jeddah, addressing knowledge gaps for improved clinical practice and future research.

Methods

This retrospective observational study was conducted from 2016 to 2021 at the pediatric oncology clinic in National Guard Hospital, Jeddah. The study focused on children aged 14 or younger at cancer diagnosis, data encompassed patient demographics, cancer details, and treatment information, including serum measurements of vitamin D (25(OH)D, calcium, phosphate, alkaline phosphatase). Vitamin D levels were categorized as deficient (<25 ng/ml), insufficient (25-49 ng/ml), sufficient (≥50- 125 ng/ml), or hypervitaminosis (>125 ng/ml), based on our center reference range and the validation of the assay.

Results

In this retrospective study of 155 pediatric oncology patients, the majority aged 0 to 10 years (78%), findings reveal a male preponderance (54.2%) and a more prevalent in patients with hematological malignancies (85%). Chemotherapy was administered to 98%, with 7% underwent radiotherapy, and 89% received steroids. Analysis of serum 25-OH vitamin D levels indicated an overall deficiency and insufficiency at diagnosis (63%) and post-therapy (43%). Age and gender had a significant influence on vitamin D levels at diagnosis, with older children and females exhibiting lower concentrations. However, these differences diminished by the end of therapy. Notably, hematological malignancy patients often presented insufficient vitamin D levels, while solid tumor patients frequently had sufficient levels. Clinical outcomes showed a high survival rate (90.7%), limited bone density assessments (18.1%), and a 14.2% prevalence of hypervitaminosis.

Conclusion

In summary, our study reveals that over two-thirds of pediatric oncology patients experience vitamin D deficiency and insufficiency at the time of diagnosis, particularly notable in females and older children. Notably, those with solid tumors exhibit higher baseline 25-OH vitamin D concentrations compared to counterparts with hematological malignancies. The findings underscore the importance of educating both patients and caregivers on supplementation and sun exposure to mitigate the prevalence of deficient and insufficient vitamin D levels in pediatric oncology cases.

## Introduction

Vitamin D is a nutrient that is required for bone health and multiple other functions; it has two forms. The first is the human-made vitamin D2 (ergocalciferol), while the second is naturally occurring vitamin D3 (cholecalciferol). Vitamin D3 is acquired through cutaneous synthesis, which is stimulated by exposure to solar ultraviolet-B radiation, producing vitamin D3. The other way is through a few dietary sources, such as oily fish and egg yolks. Vitamin D2/vitamin D3 are absorbed in the small intestines and then transformed in the liver into 25-hydroxyvitamin D2 (25(OH)D2)/25-hydroxyvitamin D3 (25(OH)D3) metabolites. Subsequently, it gets activated in the kidneys to 1,25-dihydroxyvitamin D (1,25(OH)2D) [1]. Vitamin D plays an essential role in the musculoskeletal system, as it is required for proper calcium homeostasis, appropriate growth, and skeletal development in children. Additionally, it has anti-inflammatory, immunomodulatory properties and potential cell growth regulatory capabilities [2]. This function raised the possibility of its role in cancer prevention [3,4].

Several studies have assessed the prevalence of vitamin D deficiency and insufficiency in the pediatric oncology population in the United States. Furthermore, sufficient levels of vitamin D are considered between 25 and 80 ng/mL; insufficiency is defined as 25(OH)D levels <30 ng/mL, while deficiency is agreed to be <20 ng/ mL [5-7]. A recent study has revealed that out of 163 newly diagnosed cancer patients, 52 patients were vitamin D deficient, 53 were insufficient, and 58 were sufficient. Moreover, the study indicated that females are more likely to have lower 25-hydroxy vitamin D concentrations than males [5]. In Sweden, a cross-sectional study concluded that up to 64% of the pediatric oncology population had significantly low levels of 25-hydroxyvitamin D (25(OH)D) concentration [6]. Jacmann et al. have estimated the prevalence of hypovitaminosis D among children with non-hematologic cancers; about 40.9% of children were vitamin D deficient at the time of the diagnosis and prior to treatment. In addition, they found that schoolchildren were more likely to be vitamin D deficient when compared to preschool children [6].

A systematic review has shown that vitamin D deficiency and insufficiency are more prevalent in children who have cancer compared to healthy children [8]. The reduction in vitamin D levels is attributed to several factors, including the type of cancer treatment, such as radiotherapy and chemotherapy. Radiotherapy can cause phototoxicity, so patients who receive radiotherapy are advised to avoid sunlight from where vitamin D is obtained [8]. Moreover, chemotherapy leads to hepatic and renal toxicity; as a result, vitamin D remains in its inactive form [9]. Additionally, the malignancy can decrease vitamin D concentration by reducing the body's ability to carry vitamin D to tissues. For instance, the concentration of vitamin D binding protein (DBP), an essential protein for vitamin D, along with albumin, might be reduced during the disease process. Furthermore, some anti-inflammatory drugs, such as glucocorticoids, which cancer patients take, can increase the breakdown of vitamin D. Consequently, these factors lead to the aggravation of vitamin D insufficiency [10].

Despite the significant reduction in vitamin D concentration in the pediatric oncology population, there are scarce data and knowledge gaps regarding this crucial topic [5,11]. To the best of our knowledge, no studies were conducted locally to estimate the prevalence of vitamin D deficiency in children who have cancer. Hence, this study aims to estimate the prevalence of vitamin D deficiency among the pediatric oncology population at King Abdul-Aziz Medical City (KAMC) in Jeddah between 2016 and 2021 and compare it to healthy individuals. In addition, the study aims to determine possible etiologies and to follow the changes in 25-OH vitamin D levels from the time of diagnosis and at the end of therapy. Thus, this project will help to create guidelines for managing this population and will emphasize the importance of maintaining a sufficient vitamin D level in healthy individuals as a factor that might play a role in cancer prevention.

## Materials and methods

This is a retrospective cohort study that was conducted among pediatric oncology patients selected during the period from 2016 to 2021. The study was conducted in a pediatric oncology clinic at National Guard Hospital, Jeddah. In total, 307 pediatric oncology patients were reviewed, and after applying the study exclusion criteria, 155 records were included in the analysis. This study was approved by the Institutional Review Board at King Abdullah International Medical Research Centre (KAIMRC).

Based on a non-probability consecutive sampling technique, we included children with solid tumors, hematological malignancies, and individuals 14 years of age or younger at the time of cancer diagnosis. Patients with benign hematology disorders and individuals with missing data or lost follow-up were removed.

Cases were collected from the BESTCare (hospital information system) at the National Guard hospital in Jeddah. The data included the patient's demographics as age, sex, ethnicity, cancer type (hematological or solid tumor), height, and weight (to ascertain body mass index (BMI), age at diagnosis, date of completion of all therapy, date of relapse if any, and chemotherapy protocol used). Furthermore, serum 25(OH)D concentrations were measured at diagnosis, during, and after therapy. Additionally, the season when measurements were collected and the duration and dosage of any vitamin D supplementation were recorded. Serum calcium (Ca), phosphate (PO4), and alkaline phosphatase levels (ALP) were also documented at diagnosis and after therapy.

The definition of vitamin D levels was grouped into deficiency if vitamin D level is less than <25 ng/ml, insufficient level if it is between 25-49 ng/ml, sufficient levels if vitamin D is ≥50-125 ng/ml, and hypervitaminosis if level greater than 125 ng/ml based on our center reference range and test validation.

Data analysis

All data analyses were done using the Statistical Package for Social Sciences, version 26 (SPSS, Armonk, NY: IBM Corp, USA). Mean and standard deviation (SD) were utilized for continuous data sets, and frequency and percentage were used to assess the categorical variables. Statistical tests to measure the association between vitamin D at the time of diagnosis, and after therapy in terms of the patient's demographic data have been performed using the Chi-square test and Fisher’s exact test. Two-tailed analysis with p<0.05 was used as the cutoff for statistical significance.

## Results

Patients characteristics

This retrospective study reviewed 155 pediatric oncology patients, as described in Table 1. Most of the patients were between 0 and 10 years old. More than half of the population (54.2%) were males. Most patients had low BMI at diagnosis. Most of the subjects (n=131, 85%) had hematological malignancies, and only 24 patients (16%) had solid tumors. The majority (98%) received chemotherapy, only (7%) underwent radiotherapy, whereas (89%) received steroids.

**Table 1:** Patients demographic data (n=155)

	Study data		N		%	
Age at diagnosis						
	0-5 years		60		39	
	5.1-10 years		60		39	
	10.1-15 years		35		23.1	
Sex						
	Male		84		54.2	
	Female		71		46	
Type of malignancy						
	Hematological		131		85	
	Solid		24		15.5	
Chemotherapy						
	Yes		152		98.1	
	No		3		2	
Radiotherapy					
	Yes		11		07.1	
	No		144		93	
Steroid use						
	Yes		138		89	
	No		17		11	
BMI (kg/m^2^)						
	Underweight (<5^th^ percentile)		121		78	
	Normal (5^th^-<85^th^ percentile)		28		18	
	Overweight (85^th^-<95^th^ percentile)		5		3	
	Obese (≥95^th^ percentile)		1		1	

Serum 25(OH)D levels

**Figure 1:**
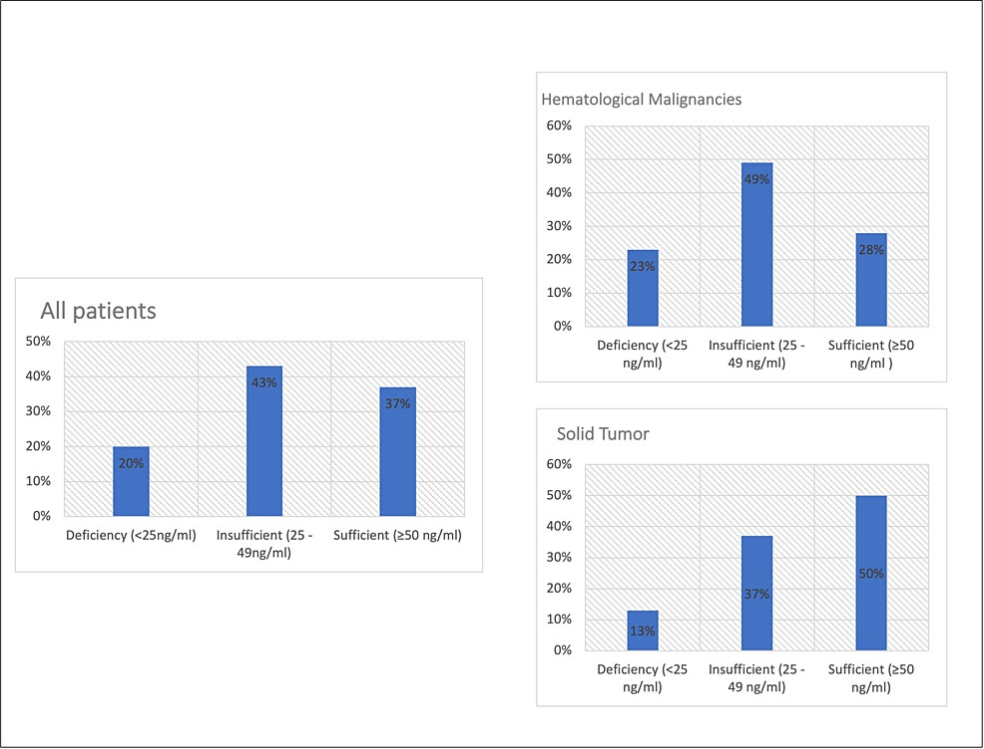
Vitamin D distribution among pediatric oncology patients at the time of diagnosis

**Figure 2:**
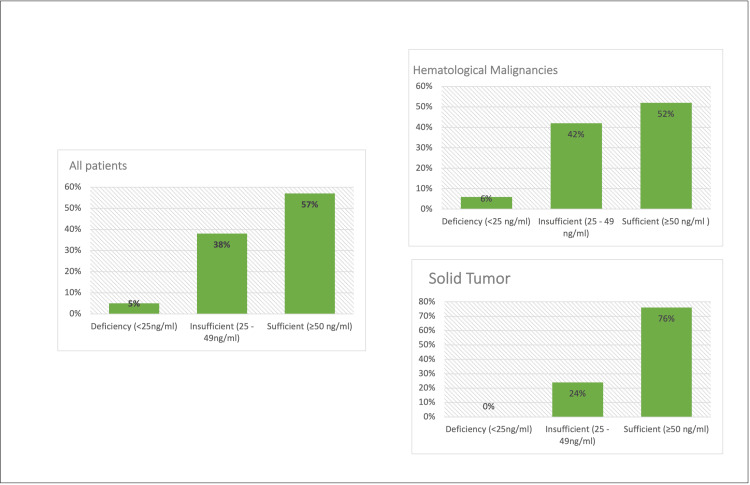
Vitamin D distribution among pediatric oncology patients at the end of therapy

The distribution of 25(OH)D levels by cancer type at the time of diagnosis and after therapy are presented in Figure 1 and Figure 2, respectively. Mean 25(OH)D levels at the time of diagnosis were 43.9 ± 26.0 ng/mL, Ca was 3.31 ± 5.19, ALP was 155.4 ± 84.6, and phosphorus was 2.28 ± 4.93 mg/dL. Thus, an overall deficiency and insufficiency of vitamin D were observed in 63% of the children at the time of diagnosis and 43% at the end of therapy.

Serum 25(OH)D levels varied significantly with age and gender at the time of diagnosis. The mean serum level of vitamin D was significantly lower among children aged 10 years and older (p = 0.001) compared with younger ages. Additionally, the female sex was associated with a lower 25(OH)D concentration compared with males, p<0.001 (Table 2). This significance does not apply to the same group of patients at the end of therapy. Sixty-four patients (49%) with hematological malignancy had insufficient levels of vitamin D. Meanwhile, 12 patients (50%) with solid tumors had sufficient levels of serum 25(OH)D at the time of diagnosis.

**Table 2:** Relationship between patients’ demographic data according to vitamin D level at the time of diagnosis and end of therapy * Patients who were lost to follow-up were excluded from the analysis. § P-value has been calculated using the Chi-square test or Fisher’s exact test. ** Significant at p<0.05 level. 25 OH = vitamin D level

Factor	Vit D at the time of diagnosis*			P-value ^§^	Vit D at the end of therapy*			
	Deficient (<25 OH ng/ml) N (%) ^(n=33)^	Insufficient (25-49 OH ng/ml) N (%) ^(n=72)^	Sufficient (≥50 OH ng/ml) N (%) ^(n=50)^		Deficient (<25 OH ng/ml) N (%) ^(n=06)^	Insufficient (<25 OH ng/ml) N (%) ^(n=55)^	Sufficient (<25 OH ng/ml) N (%) ^(n=68)^	P-value ^§^
Age at diagnosis								
0-5 years	09 (27.3%)	19 (26.4%)	32 (64.0%)	<0.001 **	03 (50.0%)	22 (40.0%)	29 (42.6%)	0.977
5.1-10 years	05 (15.2%)	40 (55.6%)	15 (30.0%)		02 (33.3%)	25 (45.5%)	28 (41.2%)	
10.1-15 years	19 (57.6%)	13 (18.1%)	03 (06.0%)		01 (16.7%)	08 (14.5%)	11 (16.2%)	
Sex								
Male	07 (21.2%)	47 (65.3%)	30 (60.0%)	<0.001 **	04 (66.7%)	30 (54.5%)	34 (50.0%)	0.690
Female	26 (78.8%)	25 (34.7%)	20 (40.0%)		02 (33.3%)	25 (45.5%)	34 (50.0%)	
Season of the diagnosis								
Summer	09 (27.3%)	26 (36.6%)	23 (47.9%)	0.230	0	17 (32.1%)	24 (35.3%)	0.147
Fall	10 (30.3%)	18 (25.4%)	08 (16.7%)		03 (50.0%)	08 (15.1%)	07 (10.3%)	
Winter	09 (27.3%)	12 (16.9%)	05 (10.4%)		01 (16.7%)	18 (34.0%)	21 (30.9%)	
Spring	05 (15.2%)	15 (21.1%)	12 (25.0%)		02 (33.3%)	10 (18.9%)	16 (23.5%)	

At the end of therapy, sufficient vitamin D values were observed in the overall population (57%), with approximately one-third of patients (38%) having insufficient vitamin D and only 5% of deficiency (Figure 2). Furthermore, almost half of the patients (52%) presented with hematological malignancy had sufficient vitamin D values, and only 6% were deficient. Most patients with solid tumors (76%) had sufficient vitamin D, and only 24% had insufficient amounts of Vitamin D.

Clinical outcomes

Clinical outcomes are summarized in Table 3. Most patients (90.79%) were alive during the last follow-up. Radiological investigations to assess bone density were done in only 18.1% of the patients. The prevalence of patients with hypervitaminosis was 14.2% due to a lack of close follow-up while on supplementation.

**Table 3:** Clinical outcome of patients (n=155)

Study variables	N	%
Status at last follow-up		
Alive	141	90.79
Relapse	05	03.23
Death	04	02.58
Unknown	05	03.23
Radiological investigation		
Yes	28	18.1
No	127	81.9
Hypervitaminosis		
Yes	22	14.2
No	133	85.8

## Discussion

The importance of vitamin D for overall health in children and adults has long been recognized. Based on previous research, it was noted that vitamin D deficiency is a significant issue in the pediatric oncology population [1-4]. In this study, we measured the prevalence of vitamin D deficiency and insufficiency in pediatric oncology patients who were diagnosed with hematological malignancies and solid tumors at the time of diagnosis and end of therapy. Additionally, we explored the possible factors associated with such a problem.

Our results demonstrate that 67% of pediatric patients with hematological malignancies and solid tumors had deficiencies or insufficiencies of vitamin D at the time of diagnosis, and that improved to 47% by the end of therapy. Iniesta et al. showed a similar high prevalence, and 64% of children with cancer have either 25(OH)D insufficiency or deficiency at the time of diagnosis [10]. In our study, the average 25(OH)D level is 43.9 ± 26.0 ng/mL at the time of diagnosis, which is higher compared to the values reported by Aristizabal et al. (27.5 ± 12.1) and Jackman et al. (23.05) [5,6]. The variation in geographical location, vitamin D supplementation standards, as well as patient characteristics, and ethnic differences among countries, could explain the gap between different research results. Therefore, each country's prevalence of vitamin D deficiency must be studied separately to create national supplementation guidelines.

In this study, we found that patients with solid tumors have better vitamin D levels at the time of diagnosis when compared to those with hematological malignancies. This finding is the opposite of what had been reported by Aristizabal P et al. [5]. This variation could be attributed to the difference in the study population and to the fact that our sample size for solid tumors was small. Thus, further research is needed to explain these two groups' differences within the same population.

Moreover, consistent with other studies, females had a higher prevalence of vitamin D deficiency than males [5,6,12,13]. In teenagers, this finding may be related to the more restricted outside activity of the females and the conservative clothing they wear that prevents exposure to the sun rays in Saudi Arabia. Also, an essential key factor could be the lack of awareness of the significance of sun exposure for bone health. Another important finding is that we noticed an inverse association between age and serum 25(OH)D levels: as age increased, serum vitamin D levels declined. Multiple studies revealed that older children are at higher risk for vitamin D inadequacy [9,11]. This could be attributed to older children having larger absolute fat mass, which alters the tissue distribution of these fat-soluble vitamins, including vitamin D. Furthermore, it is known that levels of 1,25-dihydroxyvitamin D (1,25(OH)2D) increase throughout puberty to satisfy the higher physiological demand for calcium via enhanced intestinal absorption during the pubertal growth spurt. The drop in 25(OH)D levels could happen consequently due to increased metabolism of 25(OH)D to 1,25(OH)2D [7]. The seasonal effect at the time of diagnosis did not influence vitamin D deficiency, most likely because of the weather in our area (hot most of the year and retains warm conditions during the winter, unlike other parts of Saudi Arabia).

By the end of therapy, our results demonstrated improved vitamin D levels, but still, one-third of the patients had insufficient values. There has been a huge variation in physician's practice in terms of monitoring and interventions. Beniczky et al. had shown that intensive chemotherapy during acute lymphoblastic leukemia treatment caused a further decline in osteodensitometric values in pediatric patients who already had reduced value at the time of diagnosis. Those parameters tend to improve during the maintenance phase of therapy [14]. This highlights the importance of frequent assessment and monitoring of skeletal-related side effects, especially with the significant improvements in the survival ships of childhood cancer. Age, sex, and the season had no significant effects at the end of therapy in our cohort.

To the best of our knowledge, this paper is the first of its own in Saudi Arabia that assessed 25(OH)D deficiency prevalence in pediatric oncology patients at the time of diagnosis and after therapy, and it contributes to the motive of the formulation of a national vitamin D supplementation guideline. This study is subject to several limitations. One significant limitation is the retrospective cohort study design and the lack of a control population. An additional limitation is that our study is limited to patients being treated at the National Guard Hospital in Jeddah and, therefore, is not representative of the general pediatric oncology population of Saudi Arabia. Further studies are warranted to include patients from different regions [15]. We had inconsistent longitudinal follow-ups of vitamin D after intervention and end of therapy. That fact showed the variation in clinician practice due to a lack of clear guidelines.

## Conclusions

In conclusion, our findings show that vitamin D deficiency and insufficiency affect more than two-thirds of pediatric oncology patients at the time of diagnosis, with significantly lower baseline 25(OH)D concentrations identified in females and older children. Our results also suggest that those with solid tumors seemed to have higher serum 25(OH)D concentrations at baseline compared to pediatrics with hematological malignancies. Education about supplementation and sun exposure is necessary to decrease the magnitude of deficient and insufficient vitamin D patients.
